# Severe Decline of Estimated Glomerular Filtration Rate Associates with Progressive Cognitive Deterioration in the Elderly: A Community-Based Cohort Study

**DOI:** 10.1038/srep42690

**Published:** 2017-02-17

**Authors:** Yi-Chi Chen, Shuo-Chun Weng, Jia-Sin Liu, Han-Lin Chuang, Chih-Cheng Hsu, Der-Cherng Tarng

**Affiliations:** 1 Institute of Clinical Nursing, School of Nursing, National Yang-Ming University, Taipei, Taiwan; 2 Institute of Clinical Medicine, National Yang-Ming University, Taipei, Taiwan; 3 Center for Geriatrics and Gerontology, Division of Nephrology, Department of Internal Medicine, Taichung Veterans General Hospital, Taichung, Taiwan; 4 Institute of Population Health Sciences, National Health Research Institutes, Zhunan, Taiwan; 5 Department of Health Services Administration, China Medical University, Taichung, Taiwan; 6 Department and Institutes of Physiology, National Yang-Ming University, Taipei, Taiwan; 7 Division of Nephrology, Department of Medicine, Taipei Veterans General Hospital, Taipei, Taiwan

## Abstract

Cognitive dysfunction is closely related to aging and chronic kidney disease (CKD). However, the association between renal function changes and the risk of developing cognitive impairment has not been elucidated. This longitudinal cohort study was to determine the influence of annual percentage change in estimated glomerular filtration rate (eGFR) on subsequent cognitive deterioration or death of the elderly within the community. A total of 33,654 elders with eGFR measurements were extracted from the Taipei City Elderly Health Examination Database. The Short Portable Mental Status Questionnaire was used to assess their cognitive progression at least twice during follow-up visits. Multivariable Cox regression models were used to estimate the hazard ratio (HR) for cognitive deterioration or all-cause mortality with the percentage change in eGFR. During a median follow-up of 5.4 years, the participants with severe decline in eGFR (>20% per year) had an increased risk of cognitive deterioration (HR, 1.33; 95% confidence interval [CI], 1.08–1.72) and the composite outcome (HR, 1.17; 95% CI, 1.03–1.35) when compared with those who had stable eGFR. Severe eGFR decline could be a possible predictor for cognitive deterioration or death among the elderly. Early detection of severe eGFR decline is a critical issue and needs clinical attentions.

Cognitive decline is a major health issue among the elderly, so are chronic kidney disease (CKD) complications commonly seen in the elderly^1^,^2^,^3^,^4^. Due to the slow progression, cognitive decline is often not reported in the medical history^5^. However, small vessel disease increases the risk of cognitive decline due to cerebral ischemia, in the form of silent or subclinical brain infracts or white matter lesion^1^,^2^. Clinical studies found that microvascular damage contributes to cognition change observed in the early stage of dementia^1^,^6^. Some literature has shown that cognitive impairment and dementia are prevalent in the elderly and patients with CKD^7^,^8^,^9^. In comparison, there are many similar anatomic and vasoregulatory features in the brain and kidneys. They are both low resistance end organs, exposed to high-volume blood flow, and thus are susceptible to vascular damage^1^,^2^. However, several cross-sectional and longitudinal studies yielded conflicting results on the risk of cognitive problems among elderly CKD patients. The INVADE study, and Rush Memory and Aging Project found that the elderly with moderate to severe CKD had higher risks of severe cognitive decline than the elderly with mild CKD^10^,^11^. The longitudinal studies conducted after 2009 involving large population-based cohorts, including the 3 C study^3^, which examined elderly people with moderate CKD, failed to demonstrate an association between a low baseline of estimated glomerular filtration rate (eGFR) and increased risk of cognitive decline^1^,^3^,^12^. In short, the relationship between renal function and cognitive deterioration is not yet conclusive. Because the elderly are susceptible to kidney injury^13^,^14^, and renal dysfunction is associated with cardiovascular disease and is a potential mediator of cerebrovascular disease that may lead to impaired cognition^1^,^5^,^15^, we hypothesized that renal function decline is associated with cognitive impairment among the elderly.

GFR in the remaining nephrons often is initially elevated due to glomerular hyperfiltration and hypertrophy, but as kidney disease progresses, the decline in GFR over time represents the irreversible loss of nephrons^16^,^17^. Therefore, Clinical practice guidelines propose that the magnitude of change in eGFR signals disease progression^16^,^18^,^19^. Researchers have used eGFR decline rate as an independent potential risk factor for end stage renal disease (ESRD)^20^,^21^, all-cause mortality^20^,^22^,^23^,^24^, cardiovascular mortality^24^,^25^, and coronary heart disease^24^,^25^,^26^. Some studies used different absolute eGFR decline rates to demonstrate the relationship between eGFR decline and cognitive decline^3^,^11^,^27^, but the severity and clinical significance of renal function decline may differ across starting levels of eGFR. Notably, the 3 C study found that the elderly subjects with more rapid eGFR decline were more likely to be diagnosed with vascular dementia thereafter^3^. Additional evidence shows that the risk of cognitive deterioration in patients with CKD is approximately 8–9.9% and 21.5–37% higher in cases of mild CKD^1^,^10^,^28^ and moderate CKD^1^,^10^ respectively. However, it is important to bear in mind that for a non-CKD population, the prevalence of minimal cognitive impairment was estimated to be 3.2% and age-associated cognitive decline was estimated to be 19.3%^29^. The role of an elderly’s past eGFR trajectories contributing to cognitive deterioration is less investigated and still unclear. Moreover, pathological changes of the kidney-brain axis progress gradually. We should consider the direct neuronal injury caused by uremic toxin^1^, particularly in the elderly. Longitudinal studies are thus warranted to further evaluate the role of renal function decline in elderly people for their potential cognitive deficits^2^,^7^,^9^,^15^.

However, the definitions of eGFR decline differ among studies. Most studies have investigated the absolute annualized eGFR change, but eGFR trajectories tend to be complex due to information on baseline renal function, and systematic and random effects on creatinine measures^20^,^21^. Moreover, the eGFR decline trajectories is generally nonlinearity^17^,^19^. Especially in a relatively healthy population with high eGFR, a criterion requiring percentage of change is needed to ensure the detection of small changes in eGFR^16^,^19^. Because the percentage change of eGFR varies based on the baseline of eGFR and the duration of measurement time^16^, a time-to-event endpoint according to percentage change in eGFR is simpler and easier to implement as a clinical outcome than the absolute annual eGFR change in cohort studies^17^,^20^,^21^,^24^. Thus, it is suggested that to identify clinically significant change in eGFR, the renal function change examined in longitudinal studies needs to focus on percentage change of eGFR in a time-based period^19^,^20^,^30^. To bridge the gap in the literature, we examined whether severe renal function decline was associated with cognitive deterioration or a composite endpoint (cognitive deterioration or death) after adjusting for confounding factors in a large elderly population.

## Results

### Population Characteristics

**Figure 1:**
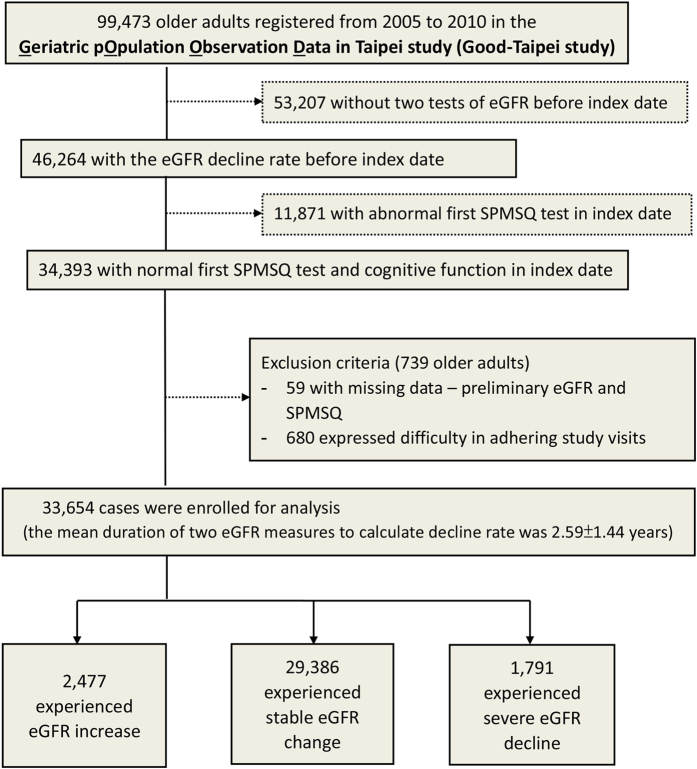
Flow chart of study patient selection. Among 33,654 elderly who had at least 2 SPMSQ and eGFR tests during the follow-up period, 2477 experienced an eGFR increase, 29,386 experienced stable eGFR changes, and 1791 experienced severe eGFR decline. The risks of cognitive deterioration and all-cause mortality were analyzed for the 3 groups.

From 99,473 older adults who participated in the annual elderly health examination in Taipei City, we enrolled 33,654 elderly individuals with normal cognitive test results and prior percentage change in eGFR on the index date. The age and gender distribution between the selected and non-selected was similar. Those were not selected mainly due to missing eGFR before index date (53,207; 53.49%) and abnormal SPMSQ (11,871; 11.93%) (Fig. 1). Participants’ characteristics by eGFR decline groups are shown in Table 1, by outcomes in Supplementary Table S1, and by CKD or not in Supplementary Table S2. The mean duration of eGFR decline measures was 2.59 (SD = 1.44) years (Fig. 1 and Supplementary Fig. S1). Most participants were male (57.20%), non-current smokers (92.90%), and non-frequent alcohol users (98.40%). Nearly half of the participants had hypertension (47.84%) and hyperlipidemia (46.26%). Most participants were non-CKD (eGFR ≥ 60, 93.63%). 84.40% of the participants had no proteinuria. In addition, laboratory measurement results showed that the participants had a relatively healthy status. The CKD group were older and had worse status in the risk factors of eGFR decline (e.g., metabolic abnormalities and proteinuria), compared to the non-CKD group (Supplementary Table S2). Among the participants, 29,386 (87.32%) of the elderly participants experienced stable eGFR change, 2,477 (7.36%) had increased eGFR, and 1,791 (5.32%) experienced a severe decline in eGFR (annual decline rate >20%) (Table 1). In the severe eGFR decline group, the mean age was 75.9 years, 59.1% were men, 51.0% had hypertension, and 53.9% had hyperlipidemia. Compared with the participants with severe eGFR decline, those who had stable eGFR or increased eGFR changes were slightly younger and more likely to be female, had less comorbidity, and had relatively good laboratory tests as well as better eGFR at baseline and a lower rate of severe proteinuria.

### Progressive Influence of eGFR Change on the Cognition of the Elderly Participants

The median follow-up of this study was 5.4 years. During the follow-ups, a total of 924 (2.8%) elderly participants had incidences of cognitive deterioration while 1,908 (5.7%) died before progressing to cognitive deterioration (Table 2). Elderly participants with cognitive deterioration had a greater number of comorbidities as well as lower serum albumin, lower total cholesterol, lower hemoglobin, lower HDL, higher white blood cell count, and relatively more severe proteinuria (Supplementary Table S1).

The incidence of cognitive deterioration increased from 5.3 to 8.2 per 1,000 person-years, indicating a 2.9 per 1,000 person-years higher incidence rate in cognitive deterioration among the severe decline group compared with the stable group (Table 2). Considering the composite outcomes of 2,832 events among the cohort, 234 (25.1%) elderly participants with severe decline in eGFR appeared to have the highest incidence rate.

**Figure 2:**
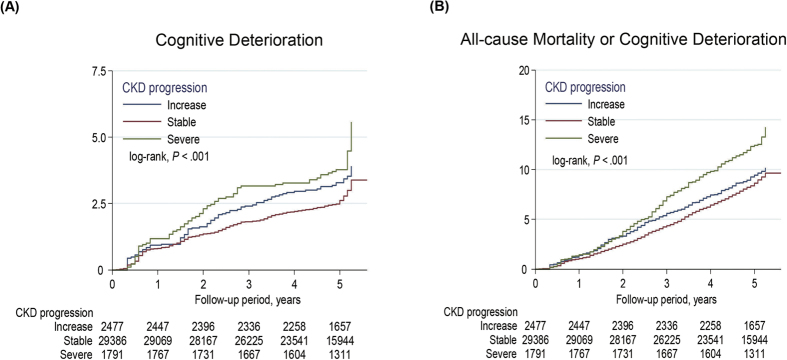
Cumulative incidence rates of (**A**) cognitive deterioration and (**B**) all-cause mortality or cognitive deterioration in the entire population are represented by the Kaplan–Meier plot.

**Figure 3:**
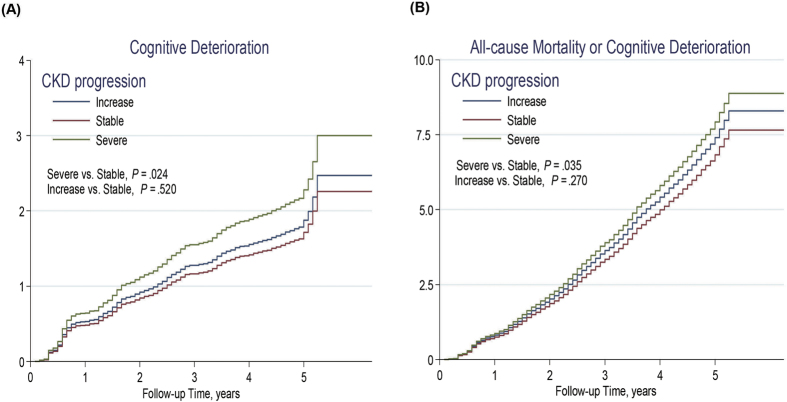
Cumulative incidence rates of (**A**) cognitive deterioration and (**B**) all-cause mortality or cognitive deterioration in the entire population are represented by the Cox proportional model.

The Kaplan–Meier plot and Cox proportional hazards model revealed that the severe eGFR decline group had a significantly increased cumulative incidence of cognitive deterioration or composite outcomes compared with the other groups (Figs 2 and 3). After adjustment for various potential confounders, the incidence rate of cognitive deterioration and composite outcomes in the severe decline group remained significantly higher than those in the other groups. Severe eGFR decline was associated with an increased risk of cognitive deterioration (adjusted hazard ratio [HR], 1.33; 95% confidence interval [CI], 1.08–1.72) and the composite outcome (adjusted HR, 1.17, 95% CI = 1.03–1.35) as compared to the stable eGFR group (Table 2). Furthermore, the cognitive deterioration incidence rate was statistically higher in the severe decline group than the stable group in the elderly with baseline eGFR < 60 ml/min per 1.73 m^2^ (adjusted HR, 1.36, 95% CI = 1.03–1.83; Supplementary Figs S2 and S3).

### Sensitivity Analysis

We found that the association between severe eGFR decline and substantial cognitive deterioration or composite outcome was similar by redefining severe decline as annual change of eGFR > 25% (HR, 1.55, 95% CI = 1.14–2.10; HR, 1.24, 95% CI = 1.08–1.49, Supplementary Table S3). The results of the severe decline as annual change of eGFR > 15% only had significantly risk increase in composite outcome (HR, 1.21, 95% CI = 1.01–1.35, Supplementary Table S4). After comparing the risk of cognitive deterioration or composite outcomes of the elderly with severe eGFR decline across different cutoff points, a tendency was identified. The greater the severity of eGFR decline, the higher the risk of cognitive deterioration and composite outcomes. Notably, when we extended the observation periods covering three measurement points, the adjusted incidence of cognitive deterioration of the severe decline group was the highest. The severe decline group had a significantly higher adjusted hazard ratio in composite outcomes when compared with the stable eGFR group (HR, 1.50, 95% CI = 1.24–1.83, Supplementary Table S5). The incidence rate of the composite outcome in the severe decline group using three measurements of eGFR was higher than the results of two measurements of eGFR before the index date (46.6 per 1,000 person-years vs.25.1 per 1,000 person-years).

The result of competing risk model analysis was also consistent in the association between eGFR decline rate and cognitive deterioration (Supplementary Table S6). It confirmed that severe eGFR decline could be a significant risk factor of cognitive deterioration when compared with the stable eGFR group.

## Discussion

To our knowledge, this is the first study with a large sample size showing severe eGFR decline is a significant factor associated with cognitive deterioration in a community elderly population. The risks of cognitive deterioration and composite outcome increased by 33% and 17% respectively, when we compared the severe eGFR decline group with stable eGFR group. The elderly with severe eGFR decline were more likely to suffer from cognitive dysfunction in the later years. We also used different cutoff points of severe eGFR decline to confirm our results. We ascertained that the annual eGFR decline >20% was a practical cutoff point indicating severe renal function decline, which can be used as a warning sign to predict cognitive deterioration and all-cause mortality in relatively healthy elderly population.

Cognitive function decline in the elderly tends to be linked with the processes of aging in the general population^2^,^14^,^31^. However, the effect of aging on memory decline may not be linear, and serum creatinine was not found to be a significant predictor of cognitive function in the elderly^32^. After 2009, the longitudinal studies have indicated that eGFR baseline levels were not related to longitudinal change in cognitive performance^3^,^27^ or ESRD risk^33^. However, the studies did not reach consensus on renal function decline as a risk factor of cognitive change. Those studies used different absolute eGFR decline rates to depict a clinical change; however, the absolute eGFR decline rates could not represent the severity of renal function progression among the population with different baseline eGFRs. Even though some studies have demonstrated that a percentage eGFR change of approximately 20–30% over 1–3 years resulted in increased risk of all-cause mortality^19^,^20^,^24^,^26^ or ESRD^19^,^20^,^33^, these studies used different follow-up durations. Clinical guidelines and previous studies usually defined a drop in eGFR of over 25% from baseline representing a significant renal function decrease^16^,^18^,^19^,^33^, but this definition cannot account for variability in creatinine related to the assay or aging. Therefore, the use of the percentage change of eGFR per year to represent renal function alteration was a better approach for clinical assessment in our outcome prediction.

Our study found that percentage change of eGFR per year is a possible influential factor of cognitive deterioration, especially in the CKD population. The 3 C study and INVADE study also showed similar results, although they used different eGFR decline rates^3^,^10^. We also demonstrated that a severe eGFR decline is related to a higher risk of cognitive deterioration in the whole study population as well as the CKD population. In our study, the CKD popuation were not only older, but also had more vascular risk factors, compared with the non-CKD population. The vascular risk factors and brain abnormalities can probably explain the high incidence of vasculopathy-related cognitive disorders in the CKD patients^1^. Moreover, the accumulation of uremic toxins may cause cerebral endothelial dysfunction and contribute to cognitive disorders in patients with CKD^34^. Therefore, declining renal function and cognition in the elderly may also be derived from a common pathophysiology^1^,^7^,^11^,^15^. Subclinical vascular disease in the kidney and brain may account for the association between cognitive dysfunction and decreased kidney function^2^,^11^.

Compared with previous studies^10^,^11^,^27^, our study had longer follow-up time. Vascular disease may cause cognitive impairment, and in cases with repeated vascular insults, it may progress to cognitive deterioration in the long term^1^,^35^. The potential key pathophysiological mechanisms underlying cognitive impairment in patients with chronic kidney damage are thought to include subclinical cerebrovascular injury^1^,^2^,^11^,^36^, endothelial dysfunction, and direct neuronal toxicity^5^,^37^,^38^, according to the renal function decline and uremic toxicity^1^,^34^,^39^. This association between renal function and impaired cognition seems to be mediated by vascular processes^2^,^3^,^7^,^35^. Severe renal damage exacerbates the uremic toxicity effect^1^,^34^,^35^, which accelerates cognitive deterioration.

In our study, the severe eGFR decline group was prone to have a higher prevalence of comorbidities. Previous study results also showed that metabolic factors not only cause renal insufficiency, but also lead to cerebrovascular disease in the elderly^1^,^3^,^9^,^15^,^40^. Davey *et al*.^27^ mentioned that the absence of comprehensive laboratory data, such as hemoglobin and albumin, was a limitation in studying the relationship between renal function and cognition. The elderly community health examination database used in our study contains detailed health-related information, including laboratory measurements (hemoglobin, albumin, and lipid profile), risky behaviors (smoking and alcohol use), comorbidities, and medication. Compared with previous studies^11^,^20^,^21^,^22^,^23^,^24^,^26^,^27^, our study suggests that severe eGFR decline would be an indicator that shows renal function change is caused by vascular effects, which echoes the hypothesis of a cognition-kidney axis.

Notably, we also discovered that the increased eGFR group had a higher incidence rate of cognitive deterioration or death. The increase in eGFR most likely indicates decreased serum creatinine as a result of muscle loss^23^,^26^,^41^ or sarcopenia^42^, particularly in the elderly. Muscle loss may be related to coexisting co-morbid diseases, malnutrition, and frailty^20^,^23^,^41^,^43^,^44^. In our study, the increased eGFR group had lower albumin and hemoglobin and more proteinuria, compared with the stable eGFR change group. The discrepancy in the increased risk of cognitive deterioration or death was found between the stable eGFR change and the increased eGFR change groups. This result could be interpreted as that the progression of cognitive deterioration and death in the elderly are not caused by increased eGFR alone. The factors leading to increased eGFR also elevate the risk of cerebrovascular and cardiovascular disease as well as mortality in community-dwelling elderly people^20^,^23^,^43^,^44^. By using sensitivity analysis, we found the elderly with continuously increasing GFR change had significantly higher rates of cognitive deterioration or death.

GFR decline is a strong and independent predictor of cardiovascular morbidity and general mortality in the elderly^14^. Our study extended the follow-up period and found that severe GFR decline caused a higher incidence of death than cognitive deterioration. The CKD patients with severe renal function decline resulted in higher risk of death. Previous studies also demonstrated that acute cardio-renal syndrome (CRS) most likely contributes to death in the elderly CKD patients^25^,^45^,^46^. They stated that CRS activates the renin-angiotensin-aldosterone system. In addition, geriatric syndromes and cardio-renal pathophysiology interfere with age-related changes in the cardiovascular system, resulting in poorly functioning kidneys^45^,^46^. However, cognitive deterioration caused by vascular injury and cerebrovascular disease may exhibit a stepwise onset. The proposed cardio-renal effect might worsen mortality rates due to severe eGFR decline^20^,^23^,^26^, and thus most elderly died before the onset of cognitive impairment in this study population.

The strengths of our study included a large sample size, longitudinal follow-up based on a clinical laboratory data, use of national death registration files, and the community-based representative nature of the investigation. Multiple factors were proposed as risk factors of cognitive deterioration. The data not only provided detailed information about comorbidities (including cardiovascular related risk factors and anemia), but also reliable laboratory examinations to demonstrate a significant association between eGFR change and health outcomes. The influence of eGFR decline on cognitive deterioration was investigated on the basis of clinically relevant changes in renal function, and this assessment was not limited to the CKD population. In addition, our study used CKD-EPI (CKD Epidemiology Collaboration) equation to estimate eGFR, which reduced the gender, race and ethnic biases. Because the study population involved in development of the MDRD formula was basically the CKD patients, which underestimate for those at higher GFR levels^16^,^17^,^30^. The CKD-EPI equation generally provides more accurate estimation, especially in non-CKD population^19^,^30^. For the community relatively healthy elderly, the CKD-EPI equation also appeared to be less biased and more accurate than the MDRD equation^17^.

This study has some limitations. First, we did not adjust for the eGFR decline during the follow-up period after the index date. The eGFR decline before and after the index date had a high correlation, thereby potentially causing multicollinearity issues, which might statistically bias the results. Second, the group with increased change in eGFR also had higher risks of cognitive deterioration or mortality, possibly as a result of muscle loss, frailty, or malnutrition^23^,^26^. However, the body composition and nutrition status of the participants were not available. In future studies, further examination of vascular injury markers and brain images will be necessary in order to explore whether uremic tonicity induced brain injuries increasingly lead to renal function progression and cognitive deterioration. Third, the large sample size might have inflated the significance of the results. The large sample size might lead to very small standard errors. This consequently causes smaller 95% confidence intervals, which allow several significant associations. The lower limits of most significant results were close to 1.0. If the sample size was smaller, these significant associations might not show up. Four, apart from missing eGFR data, the non-selected subjects had abnormal SPMSQ test at baseline as compared to the selected. However, the gender and age distribution was similar between these two groups. Accordingly, our results can only be generalized to the community elderly population who has normal SPMSQ. Finally, the SPMSQ is a short-form scale which might have underestimated the cognitive impairment among the elderly. Nevertheless, its feasibility in evaluating cognition in a large-scale elderly longitudinal cohort has been validated as well as compared with other cognitive tests, including magnetic resonance imaging^47^,^48^.

## Conclusion

In this study, a significant association between severe eGFR decline and cognitive deterioration or death was found. Furthermore, severe eGFR decline specifically contributes to a higher risk of cognitive impairment with an increased risk of 36%. Early detection of severe eGFR decline is a critical issue which is worthy to prompt clinical attention. Further studies are needed to elucidate the underlying mechanism of the association.

## Methods

### Design and Ethical Consideration

This study was designed as a retrospective longitudinal cohort study by using secondary analysis with an elderly health examination database. As every elderly participant was enrolled, his or her informed consent is obtained to authorize the Taipei City Government Institution to process health examination data for the research purpose. Detailed information on the health examination and data is stored centrally in the Taipei City Elderly Health Examination Database and is de-identified before releasing it to protect the privacy. The study was approved by the Institutional Review Board at Taipei City Hospital (TCHIRB-1010323-E), and performed in accordance with the Declaration of Helsinki.

### Data Source

The study data were obtained from the Department of Health of the Taipei City Government, which offers free annual health examination services for all citizens aged 65 years or older. Most of the elderly participants who live in community are relatively healthy than the elderly patients in long-term care institutions. The participants’ detailed characteristics in the annual health examination have been described elsewhere^49^,^50^. Briefly, health screening information from 2005 to 2010 was collected, including data on socio-demographic characteristics, date of initial registration, pre-existing comorbidities, current medication, unhealthy behaviors (smoking and alcohol use) and laboratory data. Diabetes mellitus, hypertension, and comorbidity were self-reported by the participants. A urine dipstick that tested for proteinuria was divided into negative, trace +/−, 1+, and 2+ and above. The laboratory data included total cholesterol, triglyceride, high-density lipoprotein (HDL), complete blood cell count, and serum fasting glucose.

### Selection of Participants

99,473 participants were identified from the annual elderly health examination database between March 1, 2005 and December 31, 2010 (Fig. 1). The time frame for data mining is shown in Supplementary Figure S1. For each person, the eGFR change rate with respect to the index date when the first SPMSQ tested was calculated during the analysis.

Individuals who had a normal short portable mental status questionnaire (SPMSQ) test on index dates were included. After excluding 53,207 (53.5%) elders who did not have at least 2 tests of eGFR before index dates, 11,871 (11.9%) elders who had abnormal cognitive function on the index dates at entry, 59 (0.06%) elders who had missing data, and 680 (0.7%) elders who withdrew from the study cohort, the remaining 33,645 elderly adults had received eGFR tests at least twice before the index dates. They also had normal cognitive function on index date and received at least one SPMSQ test after index date.

### Exposure Assessment

We defined eGFR decline as the percentage change in eGFR per year which was estimated by two measurements to investigate the association with subsequent cognitive deterioration. The eGFR was calculated using the CKD Epidemiology Collaboration (CKD-EPI) 2009 equation^30^,^51^. Percentage change in eGFR may be used as a clinical outcome in cohort studies^20^. Moreover, a time-to-event end point based on percentage change in estimated GFR calculated from only 2 measurements of serum creatinine is simpler and easier to implement in clinical trials than an end point defined on the rate of decline in estimated GFR^20^,^21^. The percentage change in eGFR per year was calculated by comparing the difference between two serum creatinine tests before the index date, i.e., the annual eGFR on the index date (eGFR A) and the annual eGFR prior to the annual eGFR at the index date (eGFR B). In this study, renal function change was thus defined by the change in eGFR (% change per year), which is calculated by [(eGFR A - eGFR B)/eGFR B]/follow-up interval (year)×100.

In this study, a 20% annual eGFR change was used as the cutoff point of renal function progression to provide a strict and reasonable cutoff point for clinical practice according to previous studies and clinical practice guidelines. Some studies showed that a percentage eGFR change of 15%~25% per year increased risks of ESRD or all-cause mortality^20^,^21^,^24^,^26^, but the guideline also defined a drop of greater 25% from baseline represents a clinically significant renal function decrease^16^,^18^,^19^. A meta-analysis also indicated eGFR decline in 15–20% per year could reached the observed adverse outcomes^17^. Therefore, it is reasonable to use 20% as the cutoff point to indicate severe renal function deterioration; moreover, a local study also reported that annual eGFR decline >20% was associated with a higher risk for cardiovascular and all-cause mortality in Taiwanese population^24^. We used 20% annual eGFR change as the main exposure assessment in our study. The percentage change of renal function per year was categorized into 3 groups: “severe decline” (decline in the eGFR with a >20% decrease per year), “stable” (an increase or decrease of ≤20% per year in the eGFR), and “increase” (rise in the eGFR with a >20% increase per year). Further, we have done sensitivity analyses by re-defining 15% and 25% of eGFR change as the cutoff point to validate our results.

### Outcomes

Cognitive function was measured using the SPMSQ, a tool used to briefly screen cognitive function in regular annual checkup that can be administered easily by any clinician^52^,^53^. The SPMSQ comprises 10 items to assess the presence and degree of cognitive impairment in the elderly^36^,^48^,^52^. In addition, the SPMSQ has been confirmed to be a valid tool for testing the cognitive function of Taiwanese elderly^54^. The SPMSQ scores of cognitive function as follows: a score with 0 to 2 errors indicates intact cognition, 3 to 4 errors indicates mild impairment, 5 to 7 errors indicates moderate impairment, and 8 to 10 errors indicates severe impairment^48^,^52^.

The primary end point of cognitive deterioration was ≥3 errors among the elderly on the SPMSQ after the index date to December 31, 2010. The onset of the composite outcome was the date of cognitive deterioration or all-cause mortality, whichever came first. All-cause mortality data were ascertained by linking the national death registry.

### Statistical Analysis

For baseline characteristics, continuous variables were presented as counts or percentages; descriptive results were summarized as mean ± standard deviation; and differences were tested using one-way ANOVA if the normality assumption was satisfied or using the Kruskall–Wallis test when the normal assumption was violated. For categorical variables, analyses were conducted using the Pearson χ^2^ test.

The percentage change in the eGFR/year was calculated using an ordinary least-squares regression model to prevent the over determining of equations within the magnitude of change in the eGFR^21^,^25^,^30^. Slope coefficients derived from the linear regression analysis were used to estimate the eGFR change percentage/year during the baseline period before the index date. The Cox proportional hazards model was used to show risk of cognitive deterioration and composite outcome of death and cognitive deterioration in unstable annual eGFR change after adjusting for covariates. The proportional-hazards assumption in the Cox models was not violated according to the survival plots, which were parallel in all covariates as shown in Table 1. A *P* value of <0.05 was considered significant. Different parameters such as 15% eGFR change, 25% eGFR change, and three eGFR measurements were used for sensitivity analysis to validate the consistency of our results. We also did the competing risk model analysis to confirm annual eGFR change is an independent risk factor contributing to cognitive deterioration and the composite outcome. Analyses were performed using SAS version 9.3 (SAS Institute Inc, Cary, NC, USA) and STATA SE Version 11.0 (Stata Corp, College Station, TX, USA).

## Additional Information

**How to cite this article:** Chen, Y.-C. *et al*. Severe Decline of Estimated Glomerular Filtration Rate Associates with Progressive Cognitive Deterioration in the Elderly: A Community-Based Cohort Study. *Sci. Rep.* **7**, 42690; doi: 10.1038/srep42690 (2017).

**Publisher's note:** Springer Nature remains neutral with regard to jurisdictional claims in published maps and institutional affiliations.

## Supplementary Material

Supplementary Information

## Figures and Tables

**Table 1:** Baseline Demographic and Clinical Characteristics of the Study Population.

	Increase (eGFR Change >20%)	Stable (eGFR Change −20 to 20%)	Severe Decline (eGFR Change >−20%)	*P Value*
N (%)	2,477 (7.36)	29,386 (87.32)	1,791 (5.32)	
Age, mean (SD), y	74.87 (5.60)	75.41 (5.38)	75.86 (5.63)	<0.0001
Age group, y				<0.0001
65–74	1,244 (50.22)	13,716 (46.68)	752 (41.99)	
75–84	1,103 (44.53)	13,941 (47.44)	902 (50.36)	
≥85	130 (5.25)	1,729 (5.88)	137 (7.65)	
Male	1,241 (50.10)	16,935 (57.63)	1,058 (59.07)	<0.001
Education level, y				0.194
<7	783 (31.61)	8,672 (29.51)	527 (29.42)	
7–12	858 (34.64)	10,672 (36.32)	666 (37.19)	
>12	836 (33.75)	10,042 (34.17)	598 (33.39)	
Current smoker, n (%)	164 (6.62)	2,098 (7.14)	125 (6.98)	0.616
Alcohol, n (%)	30 (1.21)	490 (1.67)	19 (1.06)	0.038
Comorbidities, n (%)				
Coronary artery disease	320 (12.92)	3853 (13.11)	242 (13.51)	0.847
Hypertension	1,172 (47.32)	14,015 (47.69)	913 (50.98)	0.023
Diabetes mellitus	199 (8.03)	2,579 (8.78)	177 (9.88)	0.109
Hyperlipidemia	1,055 (42.59)	13,550 (46.11)	965 (53.88)	<0.0001
Laboratory measurement; mean (SD)				
Serum albumin, g/dL	4.33 (0.30)	4.37 (0.29)	4.39 (0.33)	<0.0001
Glucose, mg/dL	101.30 (20.63)	104.14 (23.77)	108.14 (27.84)	<0.0001
Cholesterol, mg/dL	195.67 (33.84)	196.14 (34.16)	198.04 (35.74)	0.056
Triglyceride, mg/dL	125.26 (73.39)	121.33 (69.90)	134.02 (84.02)	<0.0001
Uric acid, mg/dL	5.80 (1.35)	6.00 (1.46)	6.52 (1.74)	<0.0001
White blood cell count, 10^3^/μL	5.70 (1.38)	5.81 (1.40)	6.04 (1.50)	<0.0001
Hemoglobin, g/dL	13.7 (1.71)	13.8 (2.51)	13.5 (1.62)	<0.0001
High-density lipoprotein (HDL), g/dL	52 (13.49)	53 (13.69)	50 (13.71)	<0.0001
Baseline eGFR, ml/min; n (%)				<0.0001
>90	65 (2.62)	1,938 (6.59)	115 (6.42)	
89–60	1,342 (54.18)	17,378 (59.14)	930 (51.93)	
45–59	877 (35.41)	8,328 (28.34)	538 (30.04)	
30–44	162 (6.54)	1,577 (5.37)	156 (8.71)	
<30	31 (1.25)	165 (0.56)	52 (2.90)	
Proteinuria, n (%)				<0.0001
Negative	2,131 (86.46)	24,760 (84.55)	1,413 (79.20)	
+/‒	141 (5.73)	2264 (7.75)	125 (7.03)	
+	112 (4.55)	1,387 (4.75)	141 (7.93)	
++ and more	80 (3.25)	861 (2.95)	104 (5.85)	

Unless otherwise indicated, data are expressed as number (percentage) of patients. Percentages have been rounded and might not total 100.

Abbreviations: CKD, chronic kidney disease; eGFR, estimated glomerular filtration rate.

**Table 2:** Incidence Rates of Cognitive Deterioration And Cognitive Deterioration or Death In the Study Population^a^.

Percentage Change in eGFR	Cognitive Deterioration Follow-up Time (years) [median (Q1-Q3)]					Study Outcome, HR (95%CI)			
		No. of Events		Incidence Rate (per 1,000 Person-years)		Cognitive Deterioration		Cognitive Deterioration or Death	
		Cognitive Deterioration	Cognitive Deterioration or Death	Cognitive Deterioration	Cognitive Deterioration or Death	Unadjusted	Adjusted	Unadjusted	Adjusted
All	5.4 (5.2–5.6)	924	2,832	5.5	17.0				
Increase (> +20%)	5.5 (5.3–5.6)	84	236	6.5	18.3	1.23 (0.98–1.54)	1.09 (0.85–1.39)	1.09 (0.95–1.25)	1.09 (0.94–1.25)
Stable (+20% to −20%)	5.4 (4.5–5.6)	763	2,362	5.3	16.4	1.0 (reference)	1.0 (reference)	1.0 (reference)	1.0 (reference)
Severe decline (> −20%)	5.5 (5.3–5.6)	77	234	8.2	25.1	1.54 (1.21–1.94)	**1.33 (1.08–1.72)**	1.49 (1.30–1.70)	**1.17 (1.03–1.35)**
Non CKD Baseline eGFR ≥60									
Increase (> +20%)	5.5 (5.3–5.6)	68	180	6.0	15.9	1.29 (1.01–1.67)	1.14 (0.56–1.51)	1.19 (1.02–1.39)	1.14 (0.96–1.35)
Stable (+ 20% to −20%)	5.4 (4.6–5.6)	249	1,318	4.6	13.0	1.0 (reference)	1.0 (reference)	1.0 (reference)	1.0 (reference)
Severe decline (> −20%)	5.6 (5.4–5.7)	14	36	4.5	11.5	0.94 (0.55–1.60)	1.28 (0.75–2.17)	0.85 (0.61–1.18)	1.05 (0.75–1.47)
CKD Baseline eGFR < 60		373	1,298						
Increase (> + 20%)	5.5 (5.3–5.6)	16	56	10.2	35.8	1.49 (0.90–2.47)	1.09 (0.62–1.91)	1.44 (1.10–1.89)	1.15 (0.86–1.53)
Stable (+ 20% to −20%)	5.4 (4.4–5.6)	294	1,044	6.9	24.4	1.0 (reference)	1.0 (reference)	1.0 (reference)	1.0 (reference)
Severe decline (> −20%)	5.5 (5.3–5.6)	63	198	10.2	31.9	1.47 (1.12–1.94)	**1.36 (1.03–1.83)**	1.27 (1.09–1.48)	1.14 (0.96–1.34)

Abbreviations: CKD, chronic kidney disease; Q, quartile; HR, hazard ratio; CI, confidence interval.

^a^The model was adjusted by age, gender, current smoking status, alcohol use, NSAID medicine use, systolic blood pressure, body mass index, coronary artery disease, hypertension, diabetes mellitus, hyperlipidemia, albumin, glucose, cholesterol, triglyceride, uric acid, white blood count, hemoglobin, high-density lipoprotein, and baseline eGFR.
