# Author Correction: Severe Decline of Estimated Glomerular Filtration Rate Associates with Progressive Cognitive Deterioration in the Elderly: A Community-Based Cohort Study

**DOI:** 10.1038/s41598-020-65655-0

**Published:** 2020-05-26

**Authors:** Yu-Chi Chen, Shuo-Chun Weng, Jia-Sin Liu, Han-Lin Chuang, Chih-Cheng Hsu, Der-Cherng Tarng

**Affiliations:** 1 Institute of Clinical Nursing, School of Nursing, National Yang-Ming University, Taipei, Taiwan; 2 Institute of Clinical Medicine, National Yang-Ming University, Taipei, Taiwan; 3 Division of Nephrology, Department of Internal Medicine, Center for Geriatrics and Gerontology, Taichung Veterans General Hospital, Taichung, Taiwan; 4 Institute of Population Health Sciences, National Health Research Institutes, Zhunan, Taiwan; 5 Department of Health Services Administration, China Medical University, Taichung, Taiwan; 6 Department and Institutes of Physiology, National Yang-Ming University, Taipei, Taiwan; 7 Division of Nephrology, Department of Medicine, Taipei Veterans General Hospital, Taipei, Taiwan

Correction to: *Scientific Reports* 10.1038/srep42690, published online 17 February 2017

This Article contains a typographical error in the spelling of the author Yu-Chi Chen, which is incorrectly given as Yi-Chi Chen.

