# Supplementary material for: Severe Decline of Estimated Glomerular Filtration Rate Associates with Progressive Cognitive Deterioration in the Elderly: A Community-Based Cohort Study
Source: Sci Rep. 2017 Feb 17;7:42690. doi: 10.1038/srep42690 (PMC5314362; doi:10.1038/srep42690)
Supplement: Supplementary Information [file srep42690-s1.pdf]

Title: Severe Decline of Estimated Glomerular Filtration Rate Associates with Progressive Cognitive Deterioration in the Elderly: A Community-Based Cohort Study

Authorship

Yi-Chi Chen, RN, PhD[1]; Shuo-Chun Weng, MD[23]; Jia-Sin Liu, MS[4]; Han-Lin Chuang, RN, MS[1]; Chih-Cheng Hsu, MD, DrPH[245]*; Der-Cherng Tarng, MD, PhD[267]*

* Corresponding Authors

Author Affiliations:

[1] Institute of Clinical Nursing, School of Nursing, National Yang-Ming University, Taipei, Taiwan

[2] Institute of Clinical Medicine, National Yang-Ming University, Taipei, Taiwan

[3] Center for Geriatrics and Gerontology, Division of Nephrology, Department of Internal Medicine, Taichung Veterans General Hospital, Taichung, Taiwan

[4] Institute of Population Health Sciences, National Health Research Institutes, Zhunan, Taiwan

[5] Department of Health Services Administration, China Medical University, Taichung, Taiwan

[6] Department and Institutes of Physiology, National Yang-Ming University, Taipei, Taiwan

[7] Division of Nephrology, Department of Medicine, Taipei Veterans General Hospital, Taipei, Taiwan.

Corresponding Authors:

Chih-Cheng Hsu, MD, DrPH.

Institute of Population Health Sciences, National Health Research Institutes.

Address: No. 35, Keyan Road, Zhunan Town, Miaoli County, 35053, Taiwan

Tel. 886-37-246166 (ext.36336) Fax: 886-37-586261 E-mail: cch@nhri.org.tw

And

Der-Cherng Tarng, MD, PhD.

Division of Nephrology, Department of Medicine, Taipei Veterans General Hospital.

Address: NO.201, Section 2, Shih-Pai Road, Taipei, 11217, Taiwan.

Tel.886-2-2826-7080 Fax: 886-2-2826-4090 E-mail: dctarng@vghtpe.gov.tw

Six Supplementary tables

Supplementary Table s1. Baseline Demographic and Clinical Characteristics of Study Population With And Without Cognitive Deterioration

Supplementary Table s2. Baseline Demographic and Clinical Characteristics of Study Population with CKD or Non-CKD

Supplementary Table s3. Incidence Rates and Risks of Cognitive Deterioration and Cognitive Deterioration or Death in The Study Population Based on 25% Change in eGFR

Supplementary Table s4. Incidence Rates and Risks of Cognitive Deterioration and Cognitive Deterioration or Death in The Study Population Based on 15% Change in eGFR

Supplementary Table s5. Risk of Cognitive Deterioration and Cognitive Deterioration or Death in an Elderly Population with Various Rates of eGFR Change Based on Three Measurements.

Supplementary Table s6. Incidence Rates and Risks of Cognitive Deterioration in the Study Population by Competing Risk Model

Three Supplementary figures

Supplement Figure s1. Study design time series

Supplementary Figure s2. Cumulative incidence of cognitive deterioration at the baseline for (A) Non- CKD and (B) CKD population are represented by the Cox proportional hazards model.

Supplementary Figure s3. Cumulative incidence of all-cause mortality or cognitive deterioration at the baseline for (A) non-CKD and (B) CKD patients are represented by the Cox -proportional hazards model.

Supplementary Table s1. Baseline Demographic and Clinical Characteristics of Study Population With And Without Cognitive Deterioration

Variables	All	Cognitive Deterioration	Non Cognitive Deterioration	
	**n = 33 654**	**n = 924**	**n = 3 2730**	***P Value***
**Age, y**				<.001
65-74	15 712 (46.69)	191 (20.67)	15 521 (47.42)	
75-84	15 946 (47.38)	531 (57.47)	15 415 (47.10)	
85+	1996 (5.93)	202 (21.86)	1794 (5.48)	
Mean (SD)	75.38 (5.40)	79.69 (6.16)	75.27(5.34)	
**Male, n (%)**	19 234 (57.15)	467 (50.54)	18 767 (57.34)	<.001
**Education level, y**				<.001
< 7	9982 (29.66)	336 (36.36)	9646 (29.47)	
7-12	12 196 (36.24)	209 (22.6)	11 987 (36.62)	
> 12	11 476 (34.10)	379 (41.02)	11 097 (33.90)	
**Current smoker, n (%)**	2387 (7.09)	46 (4.98)	2341 (7.15)	.001
**Alcohol, n (%)**	539 (1.76)	4 (0.43)	535 (1.63)	<.001
**Comorbidities**				
Coronary artery disease, n (%)	4415 (13.11)	128 (13.85)	4287 (13.10)	.50
Hypertension, n (%)	16 100 (47.84)	425 (46.00)	15 675 (47.89)	.26
Diabetes mellitus, n (%)	2955 (8.78)	123 (13.31)	2832 (8.65)	<.001
Hyperlipidemia, n (%)	15 570 (46.26)	462 (50.00)	15 108 (46.16)	.002
**Laboratory measurement**				
Serum albumin, g/dL; mean (±SD)	4.39 (0.31)	4.21 (0.38)	4.37 (0.29)	<.001
Glucose, mg/dL; mean (±SD)	104.12 (24.09)	105.15 (30.84)	104 .11 (23.59)	.32
Cholesterol, mg/dL; mean (±SD)	196.23 (33.98)	192.23 (36.56)	196.32 (34.15)	<.001
Triglyceride, mg/dL; mean (±SD)	122.17 (71.04)	130.44 (80.89)	122.07 (70.74)	<.001
Uric acid, mg/dL; mean (±SD)	6.03 (1.48)	6.88 (1.57)	5.30 (1.52)	<.001
White blood cell count ,10^3/L; mean (±SD)	5.81 (1.43)	6.08 (1.47)	5.80 (1.40)	<.001
Hemoglobin, g/dL; mean (±SD)	13.82 (2.41)	13.27 (2.14)	13.78 (2.43)	<.001
High-density lipoprotein, g/dL; mean (±SD)	52.01 (14.12)	50.09 (13.82)	52.47 (13.68)	<.001
**Baseline eGFR**				<.001
>90	2118 (6.29)	37 (4.00)	2081 (6.36)	
89-60	19 650 (58.39)	498 (53.90)	19 152 (58.52)	
45-59	9743 (28.95)	292 (31.60)	9451 (28.88)	
30-44	1895 (5.63)	85 (9.20)	1810 (5.53)	
< 30	248 (0.74)	12 (1.30)	236 (0.72)	
**Proteinuria, (%)**				<.001
Negative	28 439 (84.50)	725 (80.02)	27 579 (84.56)	
+/-	2530 (7.52)	70 (7.73)	2460 (7.54)	
+	1640 (4.87)	59 (6.51)	1581 (4.85)	
++ and more	1045 (3.11)	52 (5.74)	993 (3.04)	
**eGFR decline rate**				<.001
Increase (> +20 %)	2447 (7.27)	84 (9.09)	2393 (7.31)	
Stable (20~ (-20) %)	29 386 (87.32)	763 (82.58)	28 623 (87.45)	
Severe decline (> -20%)	1791 (5.32)	77 (8.33)	1714 (5.24)	

Supplementary Table s2. Baseline Demographic and Clinical Characteristics of Study Population with CKD or Non-CKD

Variables	All	CKD	Non CKD	
	**n = 33 654**	**n = 2134**	**n = 31 511**	***P Value***
**Age, mean (SD), y**	74.87 (5.60)	78.76(5.83)	75.16(5.30)	<.001
**Age, y**				<.001
65-74	15 712 (46.69)	548 (25.57)	15 164 (48.12)	
75-84	15 946 (47.38)	1231 (57.44)	14 715 (46.70)	
85+	1996 (5.93)	364 (16.99)	1632 (5.18)	
mean (±SD)	75.38 (5.40)	79.7 (6.2)	75.3 (5.3)	
**Male, n (%)**	19 234 (57.15)	1373 (64.07)	17 861 (56.68)	<.001
**Education level, y**				<.066
< 7	9982 (29.66)	680 (31.73)	9302 (29.52)	
7-12	12 196 (36.24)	770 (35.93)	11 426 (36.26)	
> 12	11 476 (34.10)	693 (32.34)	11 783 (34.22)	
**Current smoker, n (%)**	2387 (7.09)	147 (6.86)	2240 (7.11)	.663
**Alcohol, n (%)**	539 (1.76)	32 (1.49)	507 (1.61)	<.680
**Comorbidities**				
Coronary artery disease, n (%)	4415 (13.11)	398 (18.57)	4017 (12.75)	<.001
Hypertension, n (%)	16 100 (47.84)	1250 (58.33)	14 850 (47.13)	<.001
Diabetes mellitus, n (%)	2955 (8.78)	251 (11.71)	2704 (8.58)	<.001
Hyperlipidemia, n (%)	15 570 (46.26)	1192 (55.62)	17 378 (45.63)	<.001
**Laboratory measurement**				
Serum albumin, g/dL; mean (±SD)	4.39 (0.31)	4.32 (0.32)	4.37 (0.3)	<.001
Glucose, mg/dL; mean (±SD)	104.12 (24.09)	105.27 (23.41)	104.06 (23.84)	.024
Cholesterol, mg/dL; mean (±SD)	196.23 (33.98)	196.36 (34.17)	193.94 (35.04)	<.002
Triglyceride, mg/dL; mean (±SD)	122.17 (71.04)	137.87 (82.59)	121.24 (70.07)	<.001
Uric acid, mg/dL; mean (±SD)	6.03 (1.48)	6.96 (1.70)	5.96 (1.44)	<.001
White blood cell count ,10^3/L; mean (±SD)	5.81 (1.43)	6.09 (1.52)	5.79 (1.39)	<.001
Hemoglobin, g/dL; mean (±SD)	13.82 (2.41)	13.08 (1.93)	13.80 (2.45)	<.001
High-density lipoprotein, g/dL; mean (±SD)	52.01 (14.12)	49.01 (12.86)	52.63 (13.71)	<.001
**Proteinuria, (%)**				<.001
Negative	28 439 (84.50)	1361 (64.35)	26 862 (85.76)	
+/-	2530 (7.52)	236 (11.16)	2294 (7.32)	
+	1640 (4.87)	243 (11.49)	1397 (4.46)	
++ and more	1045 (3.11)	275 (13.00)	770 (2.46)	

Supplementary Table s3. Incidence Rates and Risks of Cognitive Deterioration and Cognitive Deterioration or Death in the Study Population Based on 25% Change in eGFR

	No. of Events		Incidence Rate (per 1000 person-years)		Study Outcome, HR (95%CI)			
					Cognitive Deterioration		Cognitive Deterioration or Death	
Percentage Change in eGFR	Cognitive Deterioration	Cognitive Deterioration or Death	Cognitive Deterioration	Cognitive Deterioration or Death	Unadjusted	Adjusted	Unadjusted	Adjusted
**All**								
Increase (> +25%)	65	168	7.5	19.5	1.43 (1.11-1.84)	1.20 (0.91-1.59)	1.15 (0.99-1.35)	1.15 (0.97-1.36)
Stable (+25 to -25 %)	809	2523	5.3	16.5	1.0 (reference)	1.0 (reference)	1.0 (reference)	1.0 (reference)
Severe decline (> -25%)	50	141	10.4	29.5	1.94 (1.46-2.59)	**1.55 (1.14-2.10**)	1.74 (1.47-2.06)	**1.24 (1.08-1.49)**
**Non CKD**								
**Baseline eGFR ≥ 60**								
Increase (> +25%)	50	125	6.5	16.3	1.41 (1.06-1.89)	1.19 (0.86-1.65)	1.22 (1.02-1.47)	1.16 (0.95-1.42)
Stable (+25 to -25 %)	491	1392	4.6	13.0	1.0 (reference)	1.0 (reference)	1.0 (reference)	1.0 (reference)
Severe decline (> -25)	10	17	7.4	12.5	1.56 (0.84-2.93)	**1.99 (1.09-3.73)**	0.93 (0.57-1.49)	**1.26 (0.78-2.04)**
**CKD**								
**Baseline eGFR < 60**								
Increase (> +25%)	15	43	16.0	45.7	2.34 (1.39-3.92)	1.65 (0.92-2.97)	1.85 (1.37-2.51)	1.39 (0.99-1.95)
Stable (+25 to (-25) %)	318	1131	6.9	24.5	1.0 (reference)	1.0 (reference)	1.0 (reference)	1.0 (reference)
Severe decline (> -25%)	40	124	11.7	36.1	1.68 (1.21-2.34)	**1.50 (1.91-1.59)**	1.44 (1.2-1.73)	**1.19 (0.98-1.46)**

Abbreviations: CKD, chronic kidney disease; HR, hazard ratio; CI, confidence interval.

[a]The model was adjusted by age, gender, current smoking status, alcohol use, systolic blood pressure, body mass index, coronary artery disease, hypertension, diabetes mellitus, hyperlipidemia, albumin, glucose, cholesterol, triglyceride, uric acid, white blood count, hemoglobin, high-density lipoprotein, and baseline CKD stage.

Supplementary Table s4. Incidence Rates of Cognitive Deterioration and Cognitive Deterioration or Death in the Study Population Based on 15% Change in eGFR [a]

Percentage Change in eGFR	No. of Events		Incidence Rate (per 1000 Person-years)		Study Outcome, HR (95%CI)			
					Cognitive Deterioration		Cognitive Deterioration or Death	
	Cognitive Deterioration	Cognitive Deterioration or Death	Cognitive Deterioration	Cognitive Deterioration or Death	Unadjusted	Adjusted	Unadjusted	Adjusted
**All**								
Increase (> +15 %)	112	324	5.8	16.7	1.10 (0.90-1.34)	0.99 (0.80-1.24)	1.01 (0.90-1.13)	1.02 (0.90-1.15)
Stable (+15 to (-15) %)	685	2105	5.3	16.2	1.0 (reference)	1.0 (reference)	1.0 (reference)	1.0 (reference)
Severe decline (> -15 %)	127	403	7.4	23.5	1.39 (1.15-1.68)	1.27 (0.99-1.49)	1.41 (1.26-1.57)	**1.21 (1.01-1.35)**
**Non CKD**								
**Baseline eGFR ≥ 60**								
Increase (> +15 %)	87	235	5.3	14.3	1.15 (0.91-1.45)	1.03 (0.80-1.32)	1.06 (0.93-1.22)	1.05 (0.91-1.22)
Stable (+15 to (-15) %)	420	1198	4.6	13.1	1.0 (reference)	1.0 (reference)	1.0 (reference)	1.0 (reference)
Severe decline (> -15 %)	44	101	5.6	12.8	1.20 (0.88-1.64)	**1.38 (1.00-1.91)**	0.95 (0.77-1.16)	1.21 (0.98-1.49)
**CKD**								
**Baseline eGFR < 60**								
Increase (>+15 %)	25	89	8.6	30.8	1.25 (0.83-1.89)	1.03 (0.66-1.62)	1.27 (1.02-1.58)	1.06 (0.84-1.34)
Stable (+15 to (-15) %)	265	907	6.9	23.6	1.0 (reference)	1.0 (reference)	1.0 (reference)	1.0 (reference)
Severe decline (> -15 %)	83	302	8.9	32.5	1.29 (1.01-1.65)	1.16 (0.89-1.51)	1.34 (1.17-1.53)	**1.18 (1.03-1.36)**

Abbreviations: CKD, chronic kidney disease; Q, quartile; HR, hazard ratio; CI, confidence interval.

[a]The model was adjusted by age, gender, current smoking status, alcohol use, systolic blood pressure, body mass index, coronary artery disease, hypertension, diabetes mellitus, hyperlipidemia, albumin, glucose, cholesterol, triglyceride, uric acid, white blood count, hemoglobin, high-density lipoprotein, and baseline CKD stages.

Supplementary Table s5. Risk of Cognitive Deterioration and Cognitive Deterioration or Death in the Study Population with Various Rates of eGFR Change Based on Three Measurements.

Percentage Change in eGFR	No. of Events		Incidence Rate (per 1000 Person-years)		Study Outcome, HR (95%CI)			
					Cognitive Deterioration		Cognitive Deterioration or Death	
	Cognitive Deterioration	Cognitive Deterioration or Death	Cognitive Deterioration	Cognitive Deterioration or Death	Unadjusted	Adjusted	Unadjusted	Adjusted
**All**								
Increase (> +20%)	52	144	7.0	19.5	1.29 (0.98-1.71)	1.16 (0.85-1.58)	1.16 (0.98-1.37)	1.24 (1.04-1.47)
Stable (+20 to -20 %)	843	2560	5.4	16.4	1.0 (reference)	1.0 (reference)	1.0 (reference)	1.0 (reference)
Severe decline (> -20%)	29	128	10.5	46.6	1.94 (1.34-2.81)	1.15 (0.77-1.72)	2.82 (2.36-3.36)	**1.50 (1.24-1.83)**
**Non CKD**								
Increase (> +20%)	37	89	6.4	15.5	1.37 (0.98-1.92)	1.25 (0.87-1.81)	1.16 (0.94-1.44)	1.23 (0.98-1.54)
Stable (+20 to -20 %)	505	1416	4.6	13.0	1.0 (reference)	1.0 (reference)	1.0 (reference)	1.0 (reference)
Severe decline (> -20%)	9	29	8.6	27.6	1.84 (0.95-3.56)	1.71 (0.89-3.32)	2.09 (1.45-3.02)	**2.04 (1.41-2.95)**
**CKD**								
Increase (> +20%)	15	55	9.2	33.8	1.27 (0.75-2.12)	1.12 (0.64-1.96)	1.36 (1.04-1.78)	1.37 (1.03-1.81)
Stable (+20 to -20 %)	338	1144	7.2	24.2	1.0 (reference)	1.0 (reference)	1.0 (reference)	1.0 (reference)
Severe decline (> -20%)	20	99	11.8	58.2	1.63 (1.04-2.56)	0.99 (0.60-1.65)	2.39 (1.95-2.94)	**1.38 (1.09-1.73)**

Abbreviations: CKD, chronic kidney disease; HR, hazard ratio; CI, confidence interval.

[a]The model was adjusted by age, gender, current smoking status, alcohol use, systolic blood pressure, body mass index, coronary artery disease, hypertension, diabetes mellitus, hyperlipidemia, albumin, glucose, cholesterol, triglyceride, uric acid, white blood count, hemoglobin, high-density lipoprotein, and baseline CKD stages.

Supplementary Table s6. Incidence Rates and Risks of Cognitive Deterioration in the Study Population by Competing Risk model

Percentage Change in eGFR	Cognitive Deterioration Follow-up Time(years) [median (Q1-Q3)]	No. of Events	Incidence Rate (per 1000 Person-years)	Study Outcome, HR (95%CI)
				Cognitive Deterioration
		Cognitive Deterioration	Cognitive Deterioration	Adjusted
**All**	5.4 (5.2-5.6)	924	5.5	
Increase (> +20%)	5.5 (5.3-5.6)	84	6.5	1.06(0.83-1.36
Stable (+20 to -20 %)	5.4 (4.5-5.6)	763	5.3	1.0 (reference)
Severe decline (> -20%)	5.5 (5.3-5.6)	77	8.2	**1.33(1.09-1.73)**
**Non CKD**				
**Baseline eGFR ≥ 60**				
Increase (> +20%)	5.5 (5.3-5.6)	68	6.0	1.12(0.84-1.48)
Stable (+20 to -20 %)	5.4 (4.6-5.6)	249	2.5	1.0 (reference)
Severe decline (> -20%)	5.6 (5.4-5.7)	14	4.5	1.28(0.75-2.18)
**CKD**				
**Baseline eGFR < 60**		373		
Increase (> +20%)	5.5 (5.3-5.6)	16	10.2	1.05(0.60-1.81)
Stable (+20 to (-20) %)	5.4 (4.4-5.6)	294	6.9	1.0 (reference)
Severe decline (> -20%)	5.5 (5.3-5.6)	63	10.2	**1.36(1.02-1.86)**

Abbreviations: CKD, chronic kidney disease; Q, quartile; HR, hazard ratio; CI, confidence interval.

[a]The model was adjusted by age, gender, current smoking status, alcohol use, systolic blood pressure, body mass index, coronary artery disease, hypertension, diabetes mellitus, hyperlipidemia, albumin, glucose, cholesterol, triglyceride, uric acid, white blood count, hemoglobin, high-density lipoprotein, and baseline CKD stages.

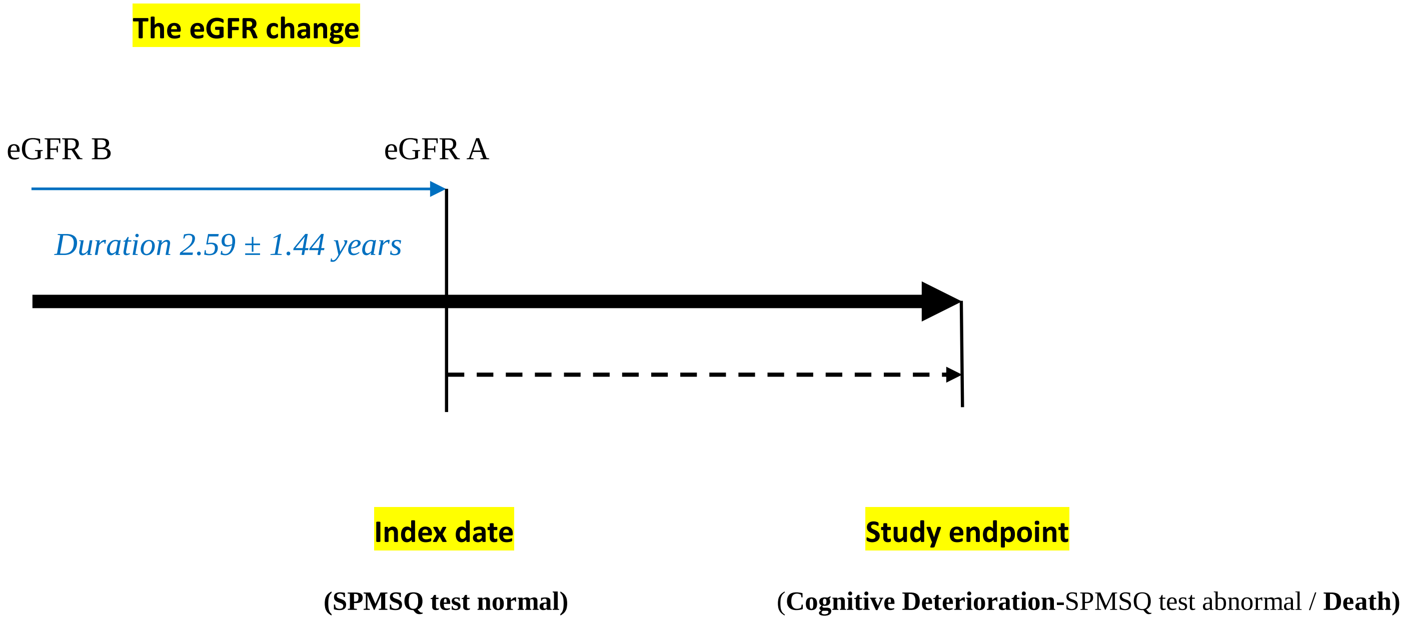

Supplement Figure s1. The time series of study design

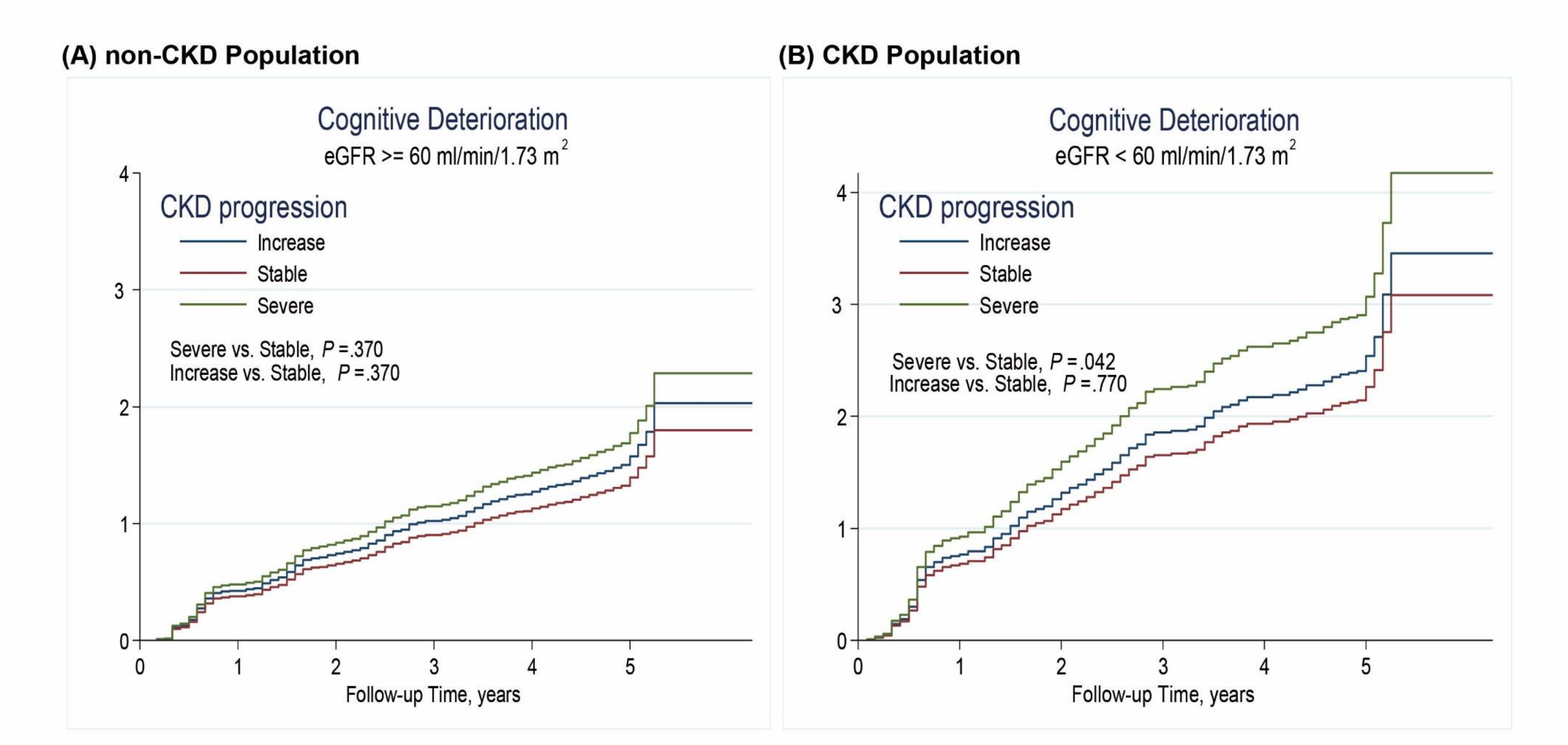

Supplementary Figure s2. Cumulative incidence of cognitive deterioration since enrollment for (A) Non- CKD and (B) CKD population are represented by the Cox proportional hazards model.

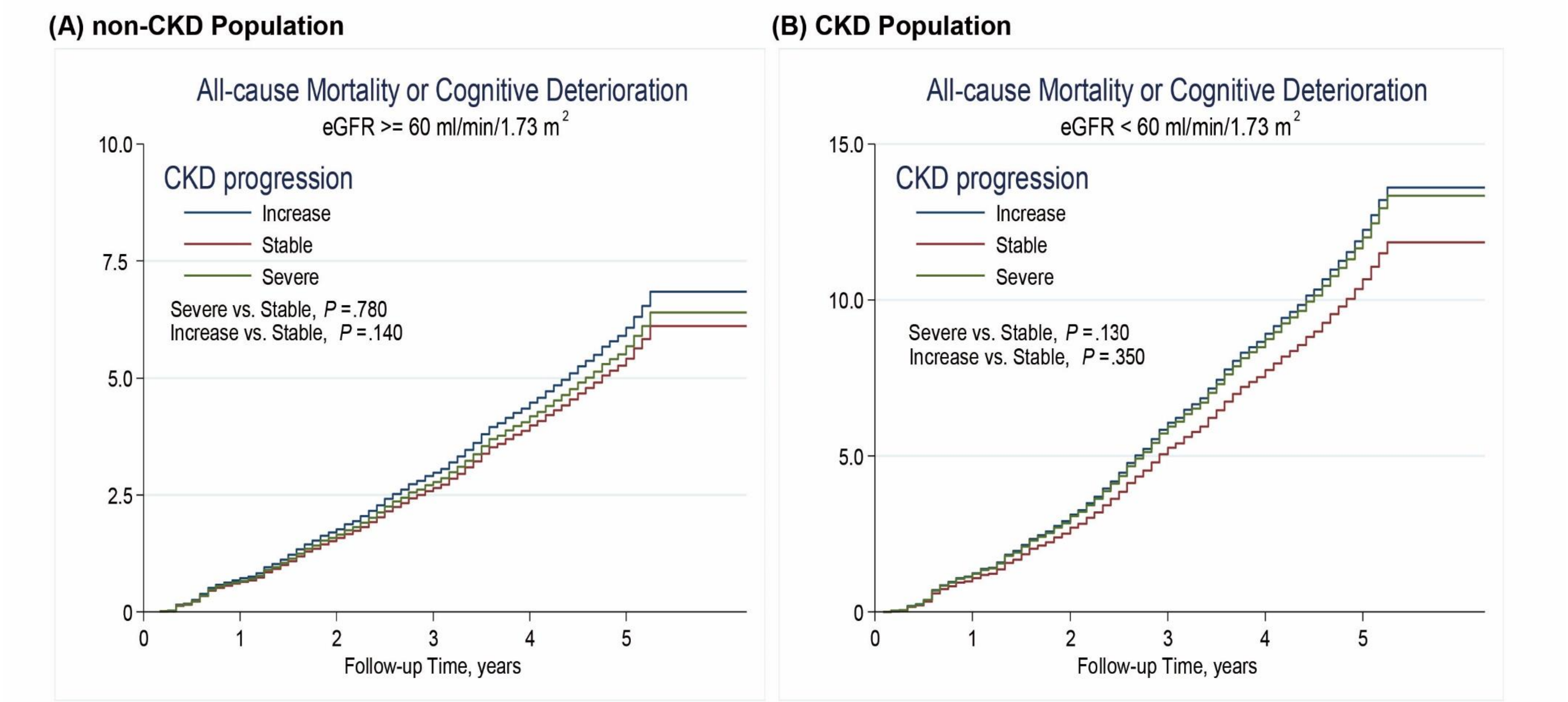

Supplementary Figure s3. Cumulative incidence of all-cause mortality or cognitive deterioration since enrollment for (A) non-CKD and (B) CKD patients are represented by the Cox -proportional hazards model.
